# Protective effect of isoflurane preconditioning on neurological function in rats with HIE

**DOI:** 10.1002/ibra.12081

**Published:** 2022-12-03

**Authors:** Yi Fei‐Sun, Miao Huang, Hao‐Yue Qin, Senio Campos de SouzaHan, Han Xue, Yu‐Ying Wang, Yi‐Bo Wang

**Affiliations:** ^1^ Institute of Neurological Disease, National‐Local Joint Engineering Research Center of Translational Medicine of Anesthesiology, West China Hospital Sichuan University Chengdu Sichuan China; ^2^ Center for Epigenetics and Induced Pluripotent Stem Cells, Kennedy Krieger Institute Johns Hopkins University Baltimore USA; ^3^ Department of Anesthesiology Southwest Medical University Luzhou Sichuan China; ^4^ State Key Laboratory of Quality Research in Chinese Medicine, Institute of Chinese Medical Sciences University of Macau Macau SAR China; ^5^ School of Basic Medical Sciences Jinzhou Medical University Jinzhou Liaoning China

**Keywords:** anesthesia, brain protection, hypoxic–ischemic encephalopathy, Isoflurane, preconditioning

## Abstract

Hypoxic–ischemic encephalopathy (HIE) is an important cause of neonatal death and disability, which can lead to long‐term neurological and motor dysfunction. Currently, inhalation anesthetics are widely used in surgery, and some studies have found that isoflurane (ISO) may have a positive effect on neuroprotection. In this paper, we investigated whether ISO pretreatment has a neuroprotective effect on the neurological function of HIE rats. Here, 7‐day‐old neonatal rats were randomly divided into a sham group, a hypoxic–ischemic (HI) group, and an ISO pretreatment (pretreatment) group. The pretreatment group was pretreated with 2% ISO for 1 h, followed by the HI group to establish an HI animal model. The HI‑induced neurological injury was evaluated by Zea‑Longa scores and triphenyltetrazolium (TTC) staining. Neuronal number and histomorphological changes were observed with Nissl staining and Hematoxylin–eosin (HE) staining. In addition, motor learning memory function was evaluated by the Morris water maze (MWM), the Y‐maze, and the rotarod tests. HI induced severe neurological dysfunction, brain infarction, and cell apoptosis as well as obvious neuron loss in neonatal rats. In the MWM, the rats in the pretreatment group showed a decrease in escape latency (*p* = 0.042), indicating that pretreatment with ISO could improve the learning ability of HI rats. The results of Nissl staining showed that in the HI group, there was an irregular arrangement of neurons and nuclear fixation; however, the cell damage was significantly reduced and the total number of neurons was increased after ISO pretreatment (*p* < 0.001). In conclusion, ISO pretreatment improved cognitive function and attenuated HI‐induced reduction of Nissl‐positive cells and spatial memory impairment, suggesting that pretreatment with ISO before HI modeling could reduce neuronal cell death in the hippocampus after HI.

## Graphical abstract

Hypoxia–ischemia (HI) causes significant brain damage, and the goal of this study was to observe if isoflurane pretreatment protects neurological function in HI rats. A 1‐h inhalation of 2% isoflurane (ISO) in rats after establishing an NHIE model resulted in neurological defects in the brain. Following this, morphological staining and behavioral tests revealed that ISO pretreatment could improve learning memory ability in rats, thereby mitigating neurological deficits.

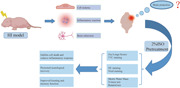

## 1 INTRODUCTION

Hypoxic–ischemic encephalopathy (HIE) is an important factor leading to neonatal death and neurodevelopmental disorders in clinical practice and is an irreversible cerebral hypoxia–ischemic (HI) injury. Its etiology includes maternal eclampsia, umbilical cord entanglement, shoulder dystocia, and placental abruption.^1^, ^2^ It can reduce or even interrupt cerebrovascular blood flow and is one of the common causes in disabled children after the neonatal period. Neonatal brain injury can result in a range of permanent neurological sequelae, including epilepsy, cerebral palsy, motor and cognitive decline, attention‐deficit hyperactivity disorder, and disability.^3^, ^4^, ^5^ Therefore, it imposes a huge mental and financial burden on families and has become one of the major medical problems worldwide. Studies have shown that about 0.2%–0.4% of newborns may present with HIE, resulting in varying degrees of cognitive and behavioral impairments in children.^6^, ^7^ Herein, an unsolved medical problem needs to be studied to elucidate the mechanism and find effective neurotherapeutic goals.^8^ At present, mild hypothermia therapy is commonly used to treat HIE in clinical practice, but 40%–50% of children with severe HIE still develop serious sequelae after treatment.^9^

Isoflurane (ISO) is an inhalation anesthetic that inhibits inflammation, prevents oxidative stress, and has some neuroprotective effects.^10^ After many years of clinical application, it has been found that it has the characteristics of reducing intracranial pressure, reducing cerebral oxygen consumption, inducing and maintaining balance, promoting rapid recovery, and inducing few side effects. Recently, studies have shown that^11^ low‐dose ISO can reduce ischemic injury, and its mechanisms of action include oxygen‐free radical injury, inflammatory response, activation of adenine receptor, activation of protein kinase C, and apoptosis and antiapoptotic mechanisms. Previous studies have shown that ISO reduces the compensatory function of one's own brain function and that inhalation of appropriate anaesthetic drugs has a preventive effect on reperfusion injury.^12^ It is closely related to postoperative cognitive dysfunction, and has the advantages of stable circulation and good muscle relaxation, which can effectively inhibit hippocampal neuronal cell apoptosis caused by cerebral ischemia–reperfusion injury, increase hypothalamic neuronal cell sodium and potassium currents, and help reduce cognitive impairment in aged rats.^13^, ^14^

**Figure 1:**
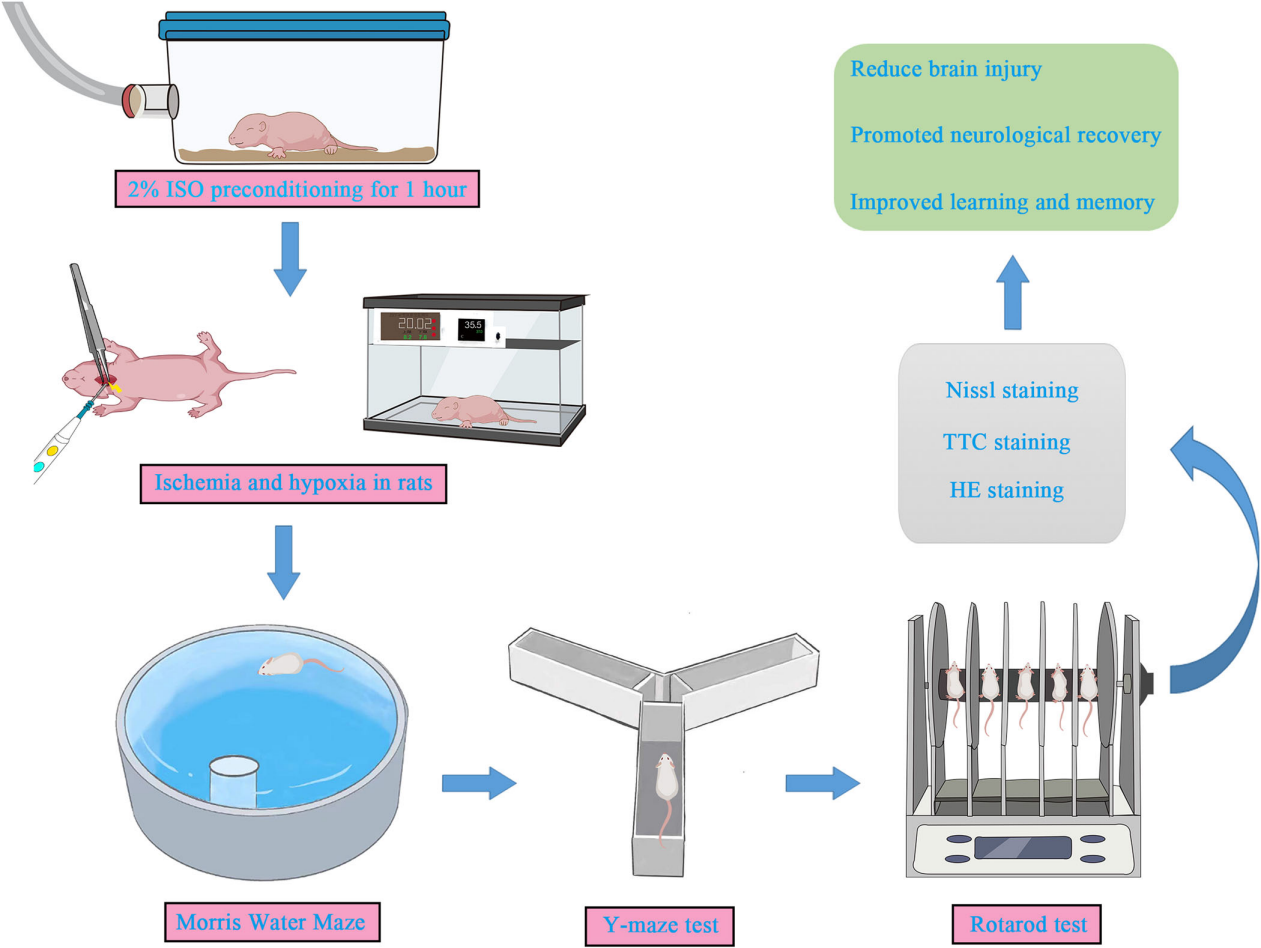
Flow chart of the study design. ISO, isoflurane; HI, hypoxia–ischemic. [Color figure can be viewed at wileyonlinelibrary.com]

In this study, we established the neonatal rat HI models and pretreated them with ISO, and behavioral and morphological experiments were performed. The present study aimed to preliminarily explore the effects of ISO preconditioning on long‐term behavioral dysfunction in HIE rats and its possible mechanism, and to provide a new therapeutic strategy for clinical treatment of HIE (Figure 1).

## 2 MATERIALS AND METHODS

### 2.1 Experimental animals and groups

The animal protocol of this study was approved by the Animal Protection and Welfare Committee of Kunming Medical University (Ethics number: Kmmu 20220748), and pregnant female SD rats used in the study were purchased from the Laboratory Animal Center of Kunming Medical University and caged individually. A total of 39 7‐day‐old rats (weighing 12–15 g) were randomly divided into a sham group, an HI group, and a pretreatment group (*n* = 13/group). All experimental procedures were performed in accordance with the procedures approved by the Animal Protection Committee of Kunming Medical University. The feeding and care of laboratory animals followed the regulations of the Chinese Committee for the Protection and Ethics of Laboratory Animals, as well as the regulations of the National Institute for Ethical Guidelines for Laboratory Animals in Health and Hygiene.

### 2.2 Establishment of the HI model

Seven‐day‐neonatal rats were anesthetized with 5% ISO (RWD Life Science Co., Ltd.) inhalation and 3% maintenance anesthesia. The rats were fixed in the supine position on the operating table and the neck was disinfected with iodophor. The right common carotid artery (RCCA) was isolated via a mid‐neckline incision and quickly ligated by electro‐cauterization (Spring Medical Beauty Equipment Co.). Finally, the rats were disinfected locally with iodophor. After 1 h of recovery, the rats were placed in a closed hypoxia chamber for 2 h, which was continuously filled with a mixture containing 6.8%–7.4% oxygen and 93.2%–92.6% nitrogen, with a gas flow rate of 1.5 L/min, temperature maintained at (33 ± 1°C), and humidity of 50%–70% to achieve hypoxia. The neonatal rats in the pretreatment group were pretreated with 2% ISO inhalation for 1 h and the sham group was only exposed to RCCA without electrocoagulation or hypoxia treatment. The vital signs of all rats needed to be closely monitored after modeling.

### 2.3 Zea‐Longa scores

The Zea‐Longa scores were applied to evaluate the neurological function of rats at 0, 5, 12, and 24 h after HI.^15^ Zea‐Longa assessment criteria are as follows: 0 point, the rat has no neurological defect; 1 point, the rat is unable to extend the affected fore limb; 2 points, the rats show contralateral rotation during walking; 3 points, the rats show contralateral falling during walking; and 4 points, the rat is unconscious and unable to walk.

### 2.4 Behavioral testing

Behavioral analysis was used to assess the functional status of the experimental animals and the nervous system. Morris water maze (MWM)^16^ and Y‐maze^17^ were used to assess the neurobehavioral function of rats, which were mainly related to spatial memory, learning, and other abilities. The rotarod test^18^ was performed to assess the motor function of rats, and severe dyskinesia or poor coordination affected the animal's ability to remain on the rotating rod when the speed was increased.

#### 2.4.1 MWM

The MWM test consisted of two parts. The spatial learning ability test was performed four times a day from 9:00 a.m. to 12:00 a.m. for 5 days, and each rat needed to be tested in different quadrants. The time of platform discovery was observed and analyzed. If the platform was not found within 90 s, it was necessary to guide the rats to the platform and stay for 20 s. Parameters such as water entry position, swimming speed, search target time, and movement trajectory were collected and analyzed using SuperMaze software (Panlab). In the spatial memory test, the platform was withdrawn and each rat was placed in the same selected quadrant (the quadrant opposite the original hidden platform) on the 6th day. The tracking software recorded the time spent in the probed quadrant and the swimming path for 1 min. During the experiment, quiet and constant light was maintained and the placement of the objects also remained unchanged. The time the rat spent in the target quadrant and the number of entries into the target quadrant were recorded as a measure of the rats’ spatial memory ability.

#### 2.4.2 Y‐maze test

The Y‐maze test was performed after the end of the MWM and the system consisted of three parts: the starting arm, the wrong arm, and the food arm. There were two main stages. The first stage was the training period. The rats were fasted 1–2 times, and their body weight decreased to 85% of the original body weight. The rats were placed in the starting arm of the instrument, and were allowed to move in the maze leaded by food, and their movement was observed and recorded. The experiment was conducted for 5 min each time, 10 min per day, for 3 consecutive days. The second session was a test session in which the wrong arm partition was withdrawn and the rat was placed by the starting arm and allowed to move freely in three arms for 5 min. The SuperMaze V2.0 system (Shanghai, Xinsoft, China) was videotaped to record the number of times the rat entered each arm and the dwell time within 5 min.

#### 2.4.3 Rotarod test

The rotarod test was used to assess motor coordination and gross motor function in rats. The rats were subjected to adaptive training 3 days before the experiment, placed on a rotarod fatigue instrument (10 rpm/min) for training three times a day for 10 min. On the third day of testing, the time of duration (seconds) on the rotarod was recorded at a constant speed of 30 rpm; the longest time among the three consecutive tests was considered as the final result.

### 2.5 Morphological staining

#### 2.5.1 Tissue harvesting

After all behavioral tests were completed on 1 and 42 days after modeling, the brain tissues of the rats were harvested. After the administration of anesthesia, the rats were fixed in the supine position and the abdominal cavity was opened to expose the thoracic cavity. Then, the heart and ascending aorta were fully exposed, a perfusion needle was inserted from the apical site, an extension tube was connected deep to the aortic root, the needle was fixed with an arterial clamp, and the right atrial appendage was cut. About 50 ml of 0.9% normal saline injection was rapidly perfused, and after the fluid flowing from the right atrial appendage became clear, slow perfusion of about 50 ml of 4% paraformaldehyde was continued until the body became stiff. The rat brain tissue was then removed, placed in a 4% paraformaldehyde solution, and fixed for 24 h for subsequent staining.

#### 2.5.2 Preparation of paraffin sections

First, the brain tissue was trimmed anteriorly from the herringbone suture into a coronal section of 4 mm, washed with running water in the cassette for 6 h, followed by dehydration with 70%, 80%, 90%, and 95% alcohol, as well as 100% alcohol I and 100% alcohol II solutions for 1 h each, and then placed in xylene I and xylene II solutions for 30 min each to make the tissue transparent. The tissues were embedded in a paraffin embedding machine (Leica model: EG1150H) and then serially sectioned into 4 μm sections. Sections were cleared and hydrated through xylene and graded alcohols for subsequent staining.

#### 2.5.3 Triphenyl tetrazolium chloride (TTC) staining

At 24 h after HI surgery, rats were anesthetized with 2.5% ISO and euthanized. Brain tissues were rapidly exposed, harvested, and photographed for anatomic appearance. Frozen brain tissues were sectioned into 2 mm coronal sections (total five slices) in a rat brain matrix before being immersed in 2% TTC solution (Sigma Co.) in a dark incubator at 37°C for 10 min. Subsequently, fixation with 4% paraformaldehyde was performed, and the infarct area was tracked and analyzed using ImageJ Software (V1.8.0.112; National Institutes of Health).

#### 2.5.4 Hematoxylin–eosin (HE) staining

The treated paraffin sections were dropped with hematoxylin staining solution (C0105; Beyotime Institute of Biotechnology) for about 3 min, and rinsed with tap water for 5 min. Then the sections were differentiated by dropping a 1% hydrochloric acid ethanol solution and they were faded for about 30 s. It was observed that the sections turned red and light in color. The sections were then returned to blue in running tap water. Eosin staining was performed for 1 min, followed by xylene clearing after dehydration from low to high gradients of alcohol. Sections were mounted by neutral gum and observed microscopically (CX40; Shunyu).

#### 2.5.5 Nissl staining

The prepared slides were dipped in Nissl staining solution (Solarbio) at room temperature for 10 min. The staining solution was decanted, and the tissue was placed in 70% alcohol for differentiation, rapidly dehydrated with absolute ethanol, cleared with xylene, and mounted with neutral gum. Three sections were randomly selected from each rat and scanned by a digital section scanner, and the percentage of neuron‐positive cells with positive Nissl staining in the hippocampus (CA1, CA2, CA3, and dentate gyrus [DG] regions) and the cortex to the total number of cells was analyzed using ImageJ software; five fields were randomly selected from each section to count the number of nerve cells with ImageJ.

### 2.6 Statistical analysis

The results were expressed as mean ± standard deviation (SD). One‐way analysis of variance (ANOVA) was used for multiple comparisons between different groups. If the data were normally distributed, the Student–Newman–Keuls post hoc test was performed. If not, Dunn's Multiple Comparison test was used to analyze the results. Escape latency was analyzed using ANOVA using repeated measures data, followed by the Bonferroni post hoc test. The relationship between morphology and behavior was determined by Pearson correlation analysis. SPSS 21.0 software (IBM Corporation, New York, NY, United States) was used for statistical analysis, and GraphPad Prism 8 software was used to draw histograms. *p* < 0.05 was considered statistically significant.

## 3 RESULTS

### 3.1 Status assessment

General manifestations of HI injury occurred in neonatal SD rats at 5–20 min of hypoxia, including cyanosis, head shaking, shortness of breath, tail pinching, limb shaking, convulsions, and other reactions; poor response, drowsiness, irregular breathing, and other phenomena persisted until the end of hypoxia after 20 min.

### 3.2 HI‐induced cerebral damage in neonatal rats

**Figure 2:**
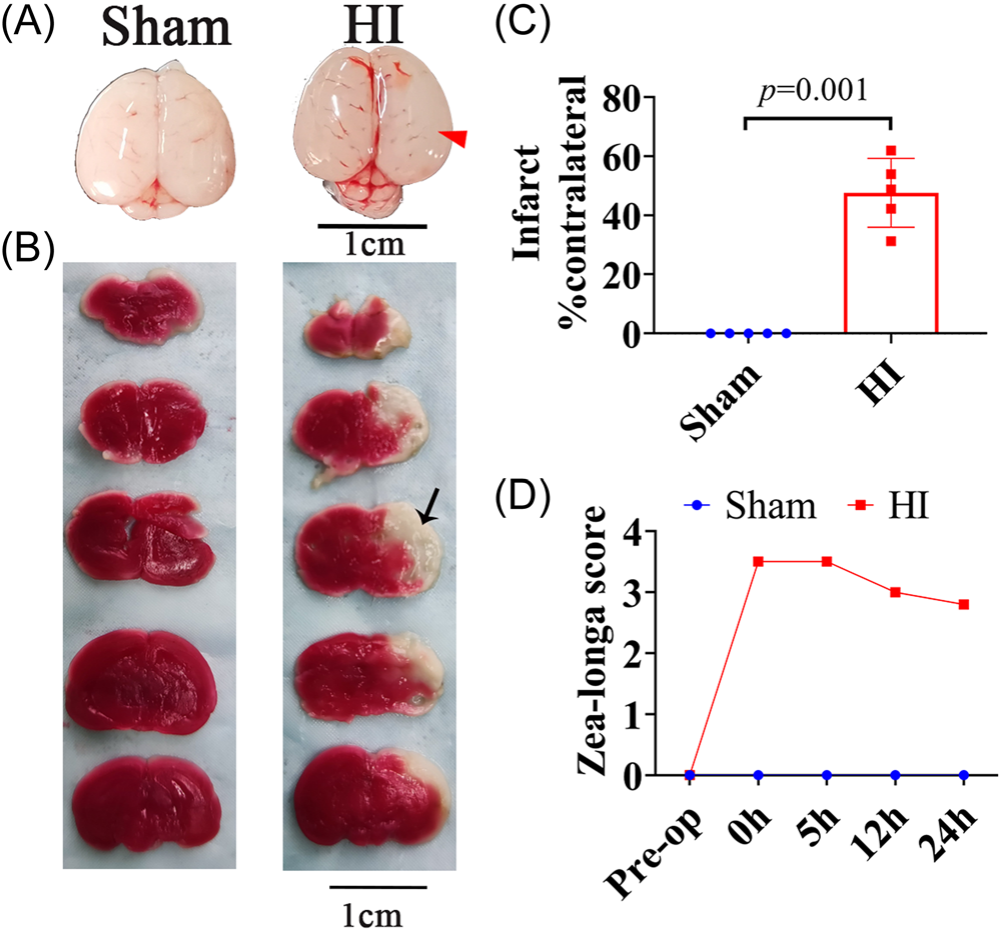
Neonatal HI‐induced brain swelling and infarction in rats. (A) Picture of the general view of the brain of sham and HI rats at 24 h after HI. The red arrow represents the swelling area. Scale bar = 1 cm. (B) TTC staining of sham and HI rats at 24 h post HI. Pale staining represents infarction areas, indicated by a black arrow. Scale bar = 1 cm. (C) Infarct ratio (% contralateral) quantified from TTC staining after HI. (D) Results of the Zea‐Longa score at 0, 5, 12, and 24 h post HI. All data are presented as mean ± SD (number of animals: *n* = 5/group). HI, hypoxia–ischemic; TTC, triphenyltetrazolium. [Color figure can be viewed at wileyonlinelibrary.com]

Brain damage was visualized on the basis of the anatomic appearance of brain tissues and TTC staining. The ipsilateral hemisphere of HI rats swelled obviously in comparison with that in the sham group (Figure 2A). Meanwhile, significant infarct was revealed in the ipsilateral hemisphere by TTC staining, but no infarct was found in rats of the sham group (Figure 2B,C, *p* = 0.001). The Zea‐Longa score of HI rats increased markedly after surgery, and showed a slowly decreasing trend within 24 h, while the scores of sham rats showed no variation at any time point (Figure 2D). These results indicated the successful establishment of HI models.

### 3.3 Pretreatment with ISO attenuated HI‐induced neurological dysfunction in neonatal rats

#### 3.3.1 Effects of ISO pretreatment on spatial learning, memory, and cognitive function in neonatal rats after HI

**Figure 3:**
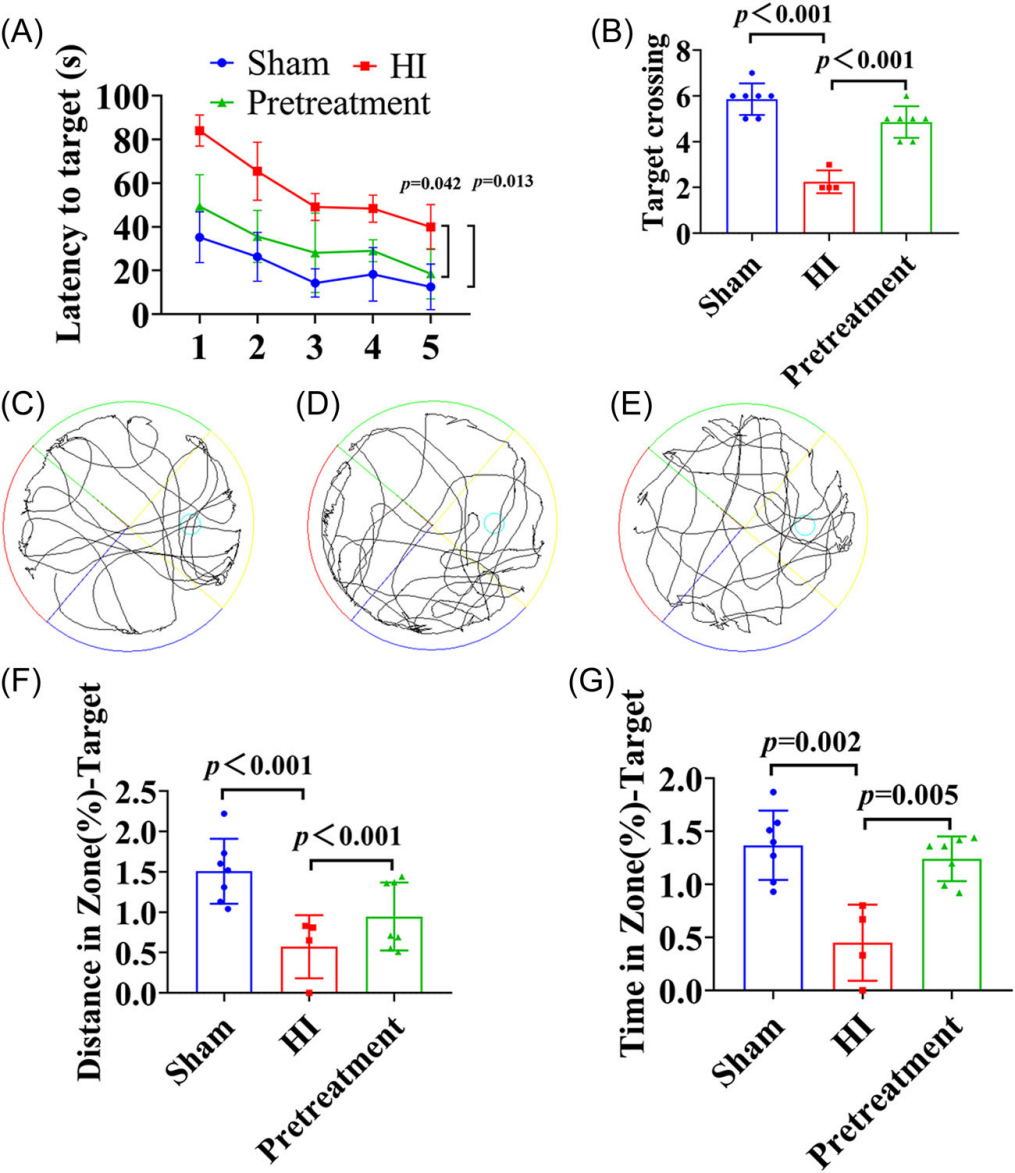
Morris water maze test in rats after isoflurane pretreatment. (A) Five‐day escape latency in rats. HI versus sham group, *p* = 0.013; pretreatment group versus HI group, *p* = 0.042. (B) Number of target platform crossings on the sixth day. (C–E) Trajectory maps of rats in the sham, HI, and pretreatment groups, respectively. (F–G) Percentage of distance and time (% = target quadrant/all quadrants). HI, hypoxia–ischemic. [Color figure can be viewed at wileyonlinelibrary.com]

The changes in the learning and memory ability of rats in each group were observed using the MWM test. The results showed that the mean escape latency of each group gradually decreased from Days 1 to 5. The escape latency was prolonged and the number of target crossings was reduced in the HI group (Figure 3A,B, *p* = 0.013, *p* < 0.001), but the escape latency was significantly decreased and the number of target crossings was increased after ISO pretreatment (Figure 3A,B, *p* = 0.042, *p* < 0.001). The movement trajectory of the three groups is shown in Figure 3C–E. The percentage of movement distance and residence time in the target quadrant were markedly reduced in the HI group (Figure 3F,G, *p* < 0.001, *p* = 0.002), but increased after the ISO pretreatment (Figure 3F,G, *p* < 0.001, *p* = 0.005).

**Figure 4:**
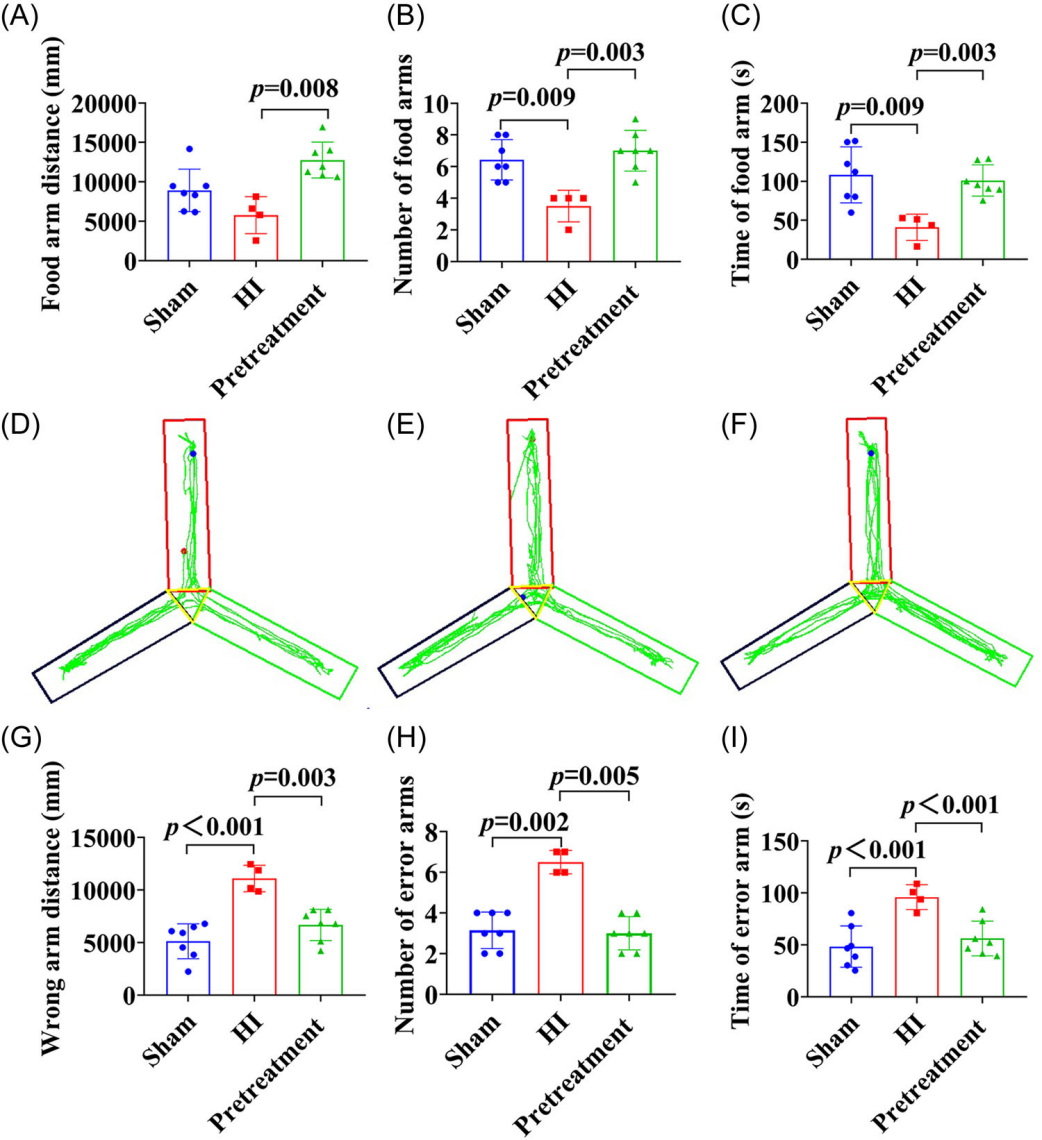
Y‐maze test in rats after isoflurane pretreatment. (A–C) Walking distance, number of entries, and duration in the food arm for sham, HI, and pretreatment rats, respectively. (D–F) Walking trajectory maps of rats in the sham, HI, and pretreatment groups, respectively. The start arm is on the top, the wrong arm is on the left, and the food arm is on the right. (G–I) Walking distance, number of entries, and duration of sham, HI, and pretreatment rats on the wrong arm, respectively.

The results of the Y‐maze test showed that the longer the walking distance, the longer the number of entries and duration in the food arm observed in rats subjected to ISO pretreatment relative to rats in the HI group (Figure 4A–C, *p* = 0.008, *p* = 0.003, *p* = 0.003). In addition, the distance of walking the wrong arm, number of entries error arms, and the residence time were increased in the HI group (Figure 4G–I, *p* < 0.001, *p* = 0.002, *p* < 0.001), which were strikingly reduced in the pretreatment group (Figure 4G–I, *p* = 0.003, *p* = 0.005, *p* < 0.001). The walking trajectory maps of rats are shown in Figure 4D–F.

#### 3.3.2 Effects of ISO pretreatment on motor function and coordination in neonatal rats after HI

**Figure 5:**
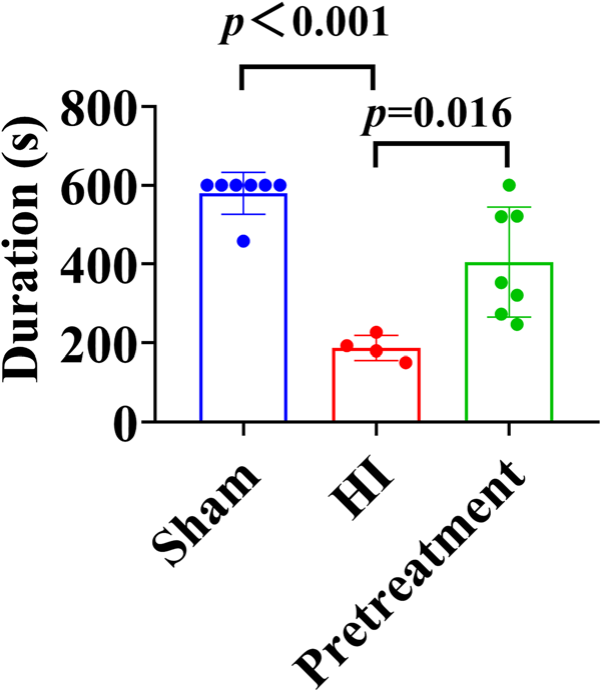
Duration of rats on the rotarod in the rotarod test. HI, hypoxia–ischemic. [Color figure can be viewed at wileyonlinelibrary.com]

To evaluate changes in motor function and coordination ability in rats after ISO pretreatment, we performed the rotarod test. In the rotarod test, the duration for which rats stayed on the rotating rod was notably shorter after HI than that of the sham group (Figure 5, *p* < 0.001), while it was longer in rats that received ISO pretreatment than rats with HI injury (Figure 5, *p* = 0.016).

### 3.4 ISO ameliorated the acute cerebral injury induced by HI in neonatal rats

**Figure 6:**
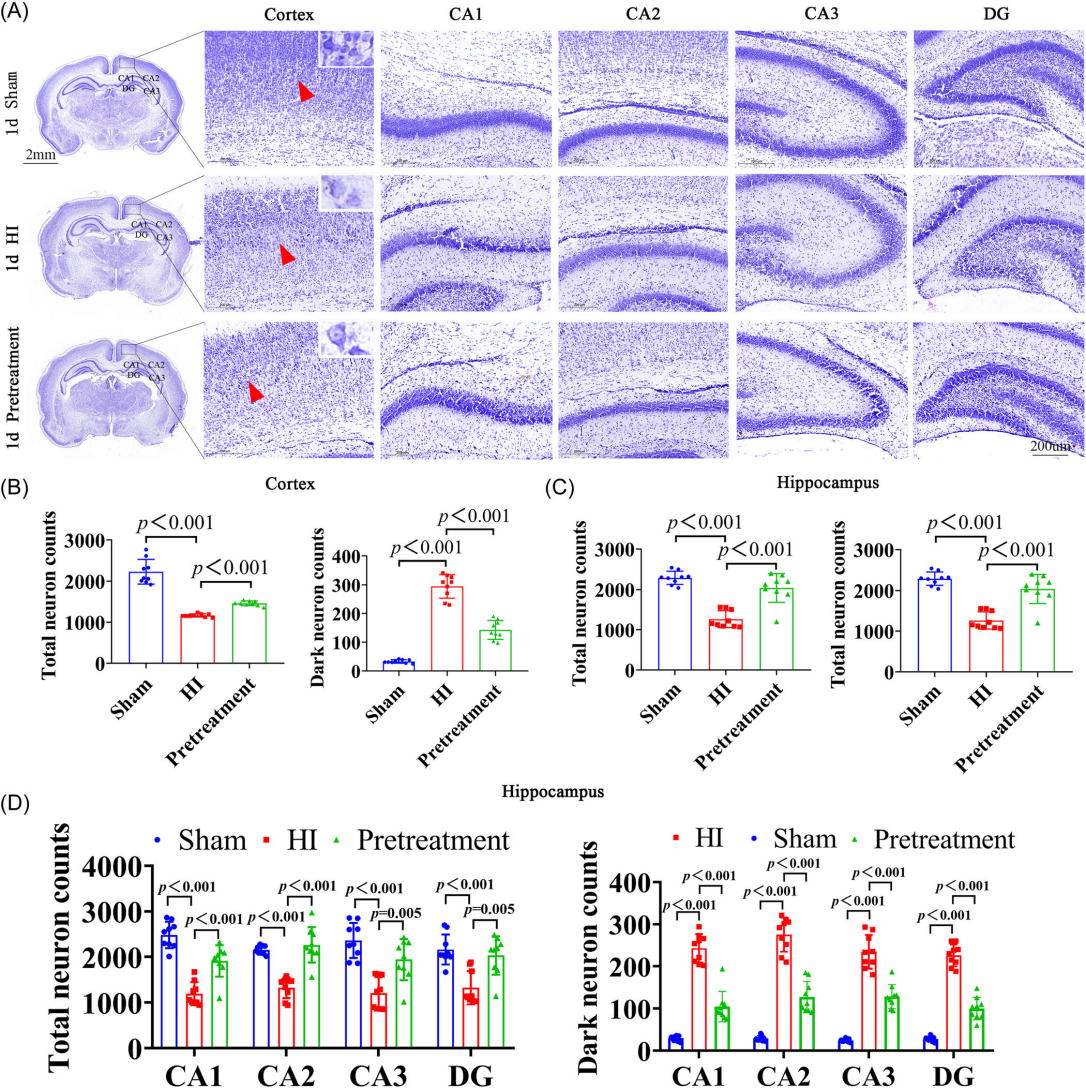
Nissl staining of ISO pretreated neonatal rats on Day 1 after HI. (A) Three images on the left show the appearance of the brains of the sham, HI, and pretreatment groups. Scale bar = 2 mm. The black box represents the right cortex and the hippocampus CA1, CA2, CA3, and DG. Scale bar = 200 μm. The red arrow shows the cell. (B) Number of total neurons in the sham, HI, and pretreatment groups in the cortex and the hippocampus. (C) Number of dark neurons in the sham, HI, and pretreatment groups in the cortex and the hippocampus. Scale bar = 200 μm. (D) Quantitative histograms of total neurons and dark neurons in the hippocampal CA1, CA2, CA3, and DG. Data are presented as mean ± SD. HI, hypoxia–ischemic; ISO, isoflurane. [Color figure can be viewed at wileyonlinelibrary.com]

To demonstrate the effect of HI on neuronal survival in the brain, Nissl staining was performed on Day 1 after modeling to analyze neuronal cell survival in this model. Results of Nissl staining showed that in the sham group, the cells of each layer were neatly arranged with abundant Nissl bodies and clearly visible oval nucleoli. Cells in the HI group demonstrated disordered cell arrangement, neuronal necrosis, and reduced Nissl bodies. However, in the pretreatment group, the number of necrotic neurons decreased, and Nissl bodies were significantly increased (Figure 6A). Compared with the sham group, the total neurons in the cortex (Figure 6B, *p* < 0.001) and the hippocampus (Figure 6C, *p* < 0.001) were significantly decreased in the HI group, which was decreased after ISO pretreatment (Figure 6B,C, *p* < 0.001, *p* < 0.001). Moreover, there were more total neurons in the CA1, CA2, CA3, and DG regions of the hippocampus and fewer dark neurons in the pretreatment group compared to the HI group (Figure 6D, *p* < 0.01).

**Figure 7:**
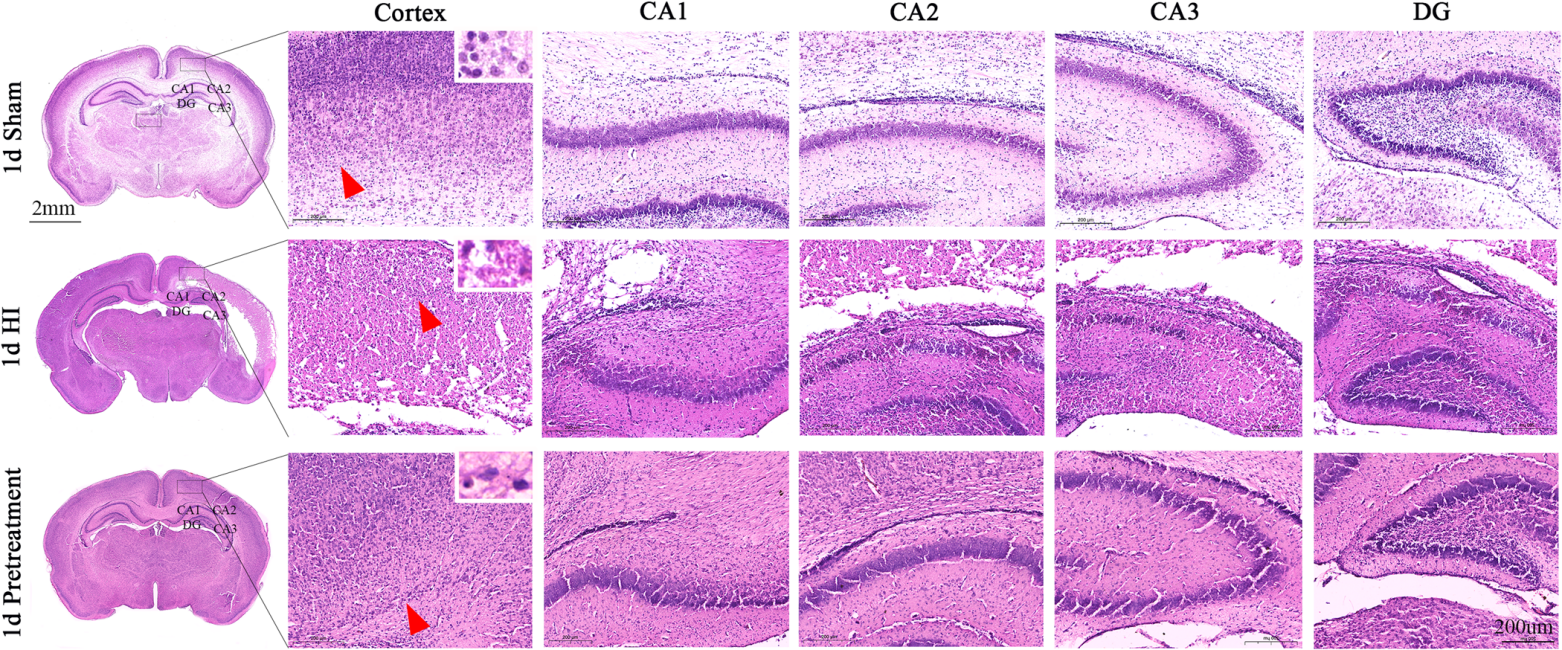
HE staining of ISO pretreated neonatal rats on Day 1 after HI. (A) Three images on the left show the appearance of the brains of the sham, HI, and pretreatment groups. Scale bar = 2 mm. The black box represents the right cortex and the hippocampus. Scale bar = 200 μm. HE, hematoxylin and eosin; HI, hypoxia–ischemic; ISO, isoflurane. [Color figure can be viewed at wileyonlinelibrary.com]

As shown by HE staining, the right brain structure of the sham rats was intact, and the various nerve cells with normal shapes had a complete structure. The nucleus is located at the center of the cell body, with a clear nuclear membrane and obvious nucleoli. However, the brain tissues in the HI group were denatured and necrotic, where cortical cells were disordered and fragmented. Many vacuoles were formed. Neuronal and cellular structures disappeared, and a large number of inflammatory cells had infiltrated. However, after ISO pretreatment, there was an obvious improvement in the disordered cell arrangement in the right cerebral cortex, and the morphology of neurons was close to that of the normal ones in the sham group (Figure 7).

### 3.5 ISO treatment ameliorated cerebral pathological changes in neonatal HI rats

**Figure 8:**
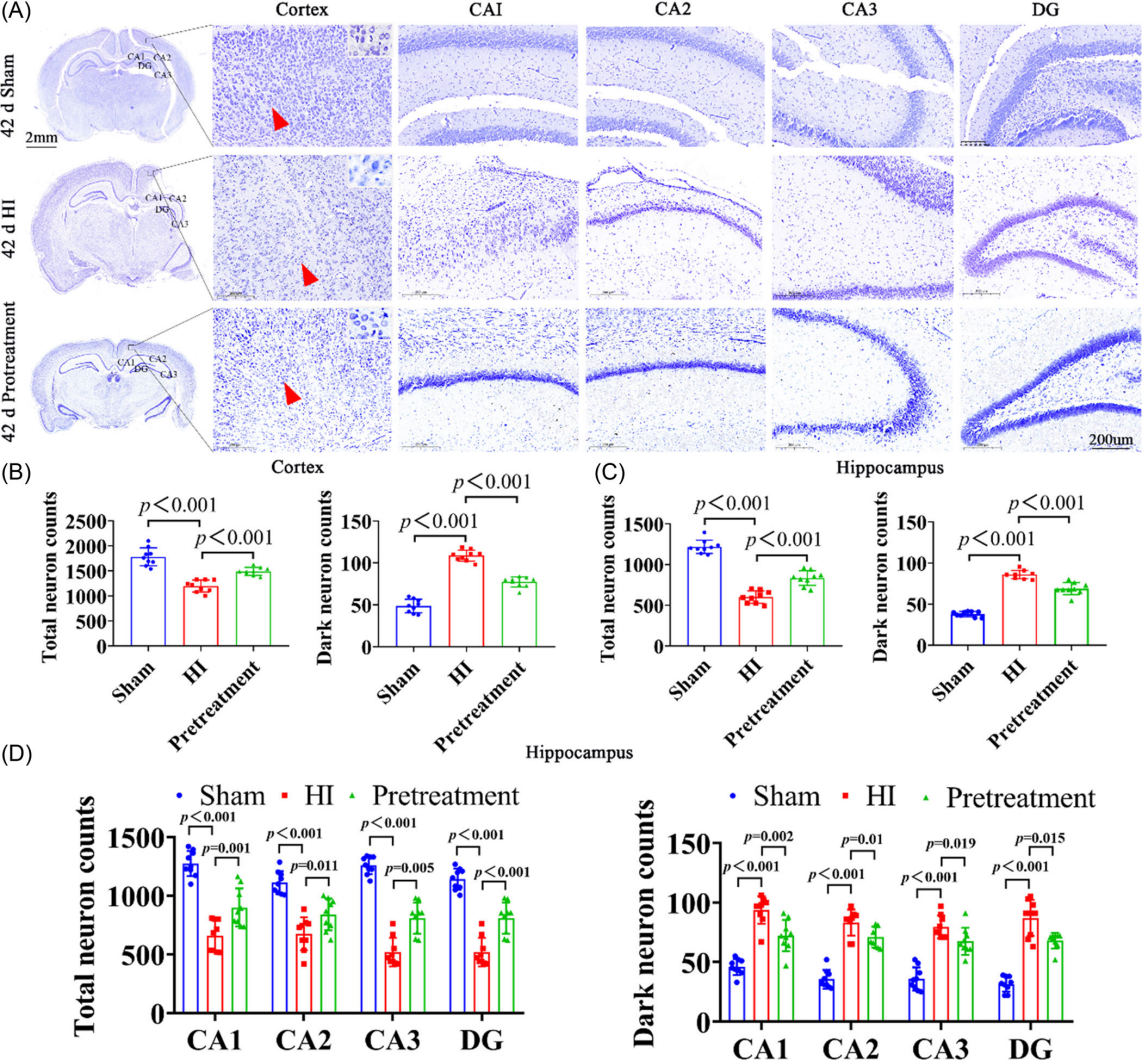
Nissl staining of ISO pretreated neonatal rats on Day 1 after HI. (A) Three images on the left show the appearance of the brains of the sham, HI, and pretreatment groups. Scale bar = 2 mm. The black box represents the right cortex and hippocampus CA1, CA2, CA3, and DG. Scale bar = 200 μm. The red arrow shows the cell. (B) Number of total neurons in the sham, HI, and pretreatment groups in the cortex and the hippocampus. (C) Number of dark neurons in the sham, HI, and pretreatment groups in the cortex and the hippocampus. Scale bar = 200 μm. (D) Quantitative histograms of total neurons and dark neurons in the hippocampal CA1, CA2, CA3, and DG. Data are presented as mean ± SD. HI, hypoxia–ischemic; ISO, isoflurane. [Color figure can be viewed at wileyonlinelibrary.com]

Nissl staining was used to detect the survival of neurons, and as a result, Nissl‐stained neurons were detected in both cortical and hippocampal regions 42d after HI. In the sham group, the neurons had an intact morphological structure, large, neatly arranged cell bodies, uniform staining, and abundant cytoplasm and Nissl bodies; however, in the HI group, the neurons were irregularly arranged and showed extensive neuronal damage, such as pyknosis, increased space, decreased volume, dark blue nuclei, interstitial swelling, neuronal structure, and disappearance of Nissl bodies (Figure 8A). Similar to the 1d results, the number of total neurons decreased significantly after HI, and the number of dark neurons markedly increased compared with the sham group, and the difference was statistically significant (Figure 8B, *p* < 0.001; Figure 8C, *p* < 0.001). Compared with the HI group, the pretreatment group showed an increased number of total neurons in the CA1, CA2, CA3, and DG of the hippocampus regions and cortical regions (Figure 8D, *p* = 0.001, *p* = 0.011, *p* = 0.005, *p* < 0.003) and a decreased number of dark neurons (Figure 8D, *p* = 0.001, *p* = 0.01, *p* = 0.019, *p* = 0.015).

**Figure 9:**
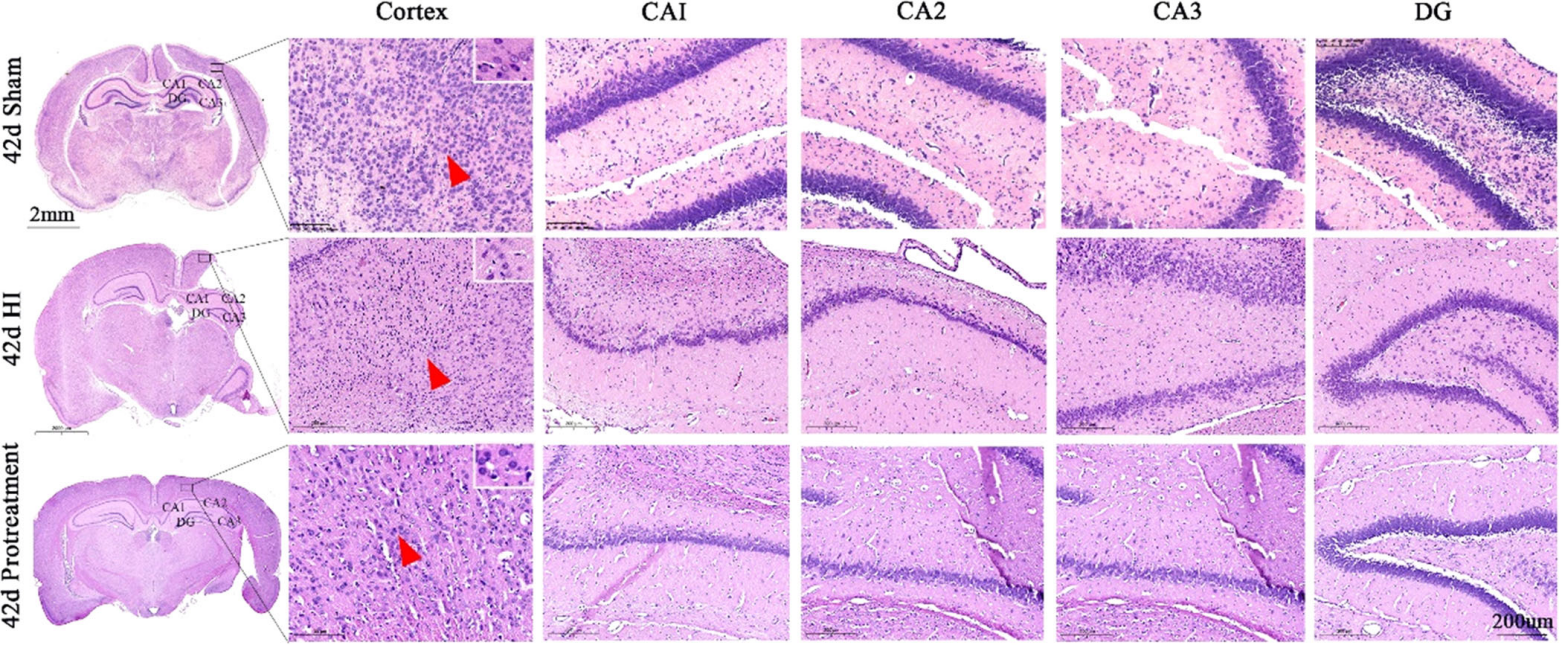
HE staining of ISO pretreated neonatal rats on Day 1 after HI. (A) Three images on the left show the appearance of the brains of sham, HI, and pretreatment groups. Scale bar = 2 mm. The black box represents the right cortex and the hippocampus. Scale bar = 200 μm. HE, hematoxylin and eosin; HI, hypoxia–ischemic; ISO, isoflurane. [Color figure can be viewed at wileyonlinelibrary.com]

HE staining was used to observe the morphological changes in brain tissue at 42 days, and the hippocampal tissue morphology of rats in the sham group was normal, the space around the cells and microvessels was normal, the nuclei were large and round, and the nuclear chromatin was uniform and clear; compared with the sham group, more cell cavities appeared in the cortex, CA1, CA2, CA3, and DG regions of the hippocampus after HI, the nuclei were pyknotic, irregularly arranged, and pressed to one side of the neuronal cells, the brain swelling was more severe, the chromatin of gliotic nuclei was blurred, the space around the cells and microvessels was significantly widened, the tissue space was significantly swollen, and the nuclei were pyknotic, densely stained, or even ruptured. However, after treatment with ISO, the cell morphology in the cortical hippocampus was more complete than that in the HI group, with less nucleolus reduction (Figure 9). These results suggested that ISO could alleviate cell damage in neonatal HI rats.

### 3.6 Correlation analysis between behavior and morphology in HI neonatal rats

**Figure 10:**
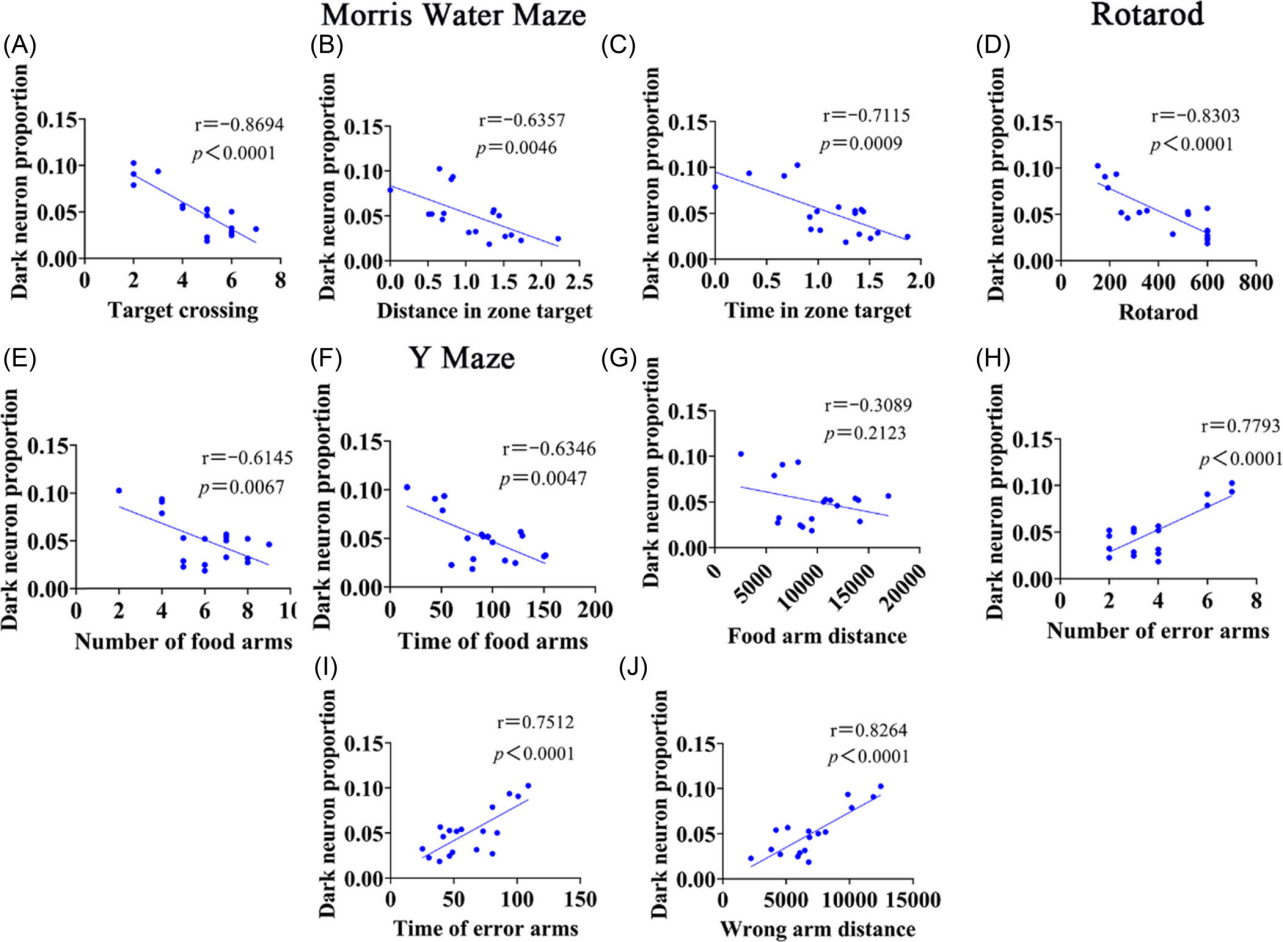
Correlation analysis between the percentage of dark neuron, a behavioral index, and each index of morphology in HI rats after isoflurane pretreatment. (A–C) Correlation analysis between morphology and the number of passes, the percentage of distance from the target quadrant, and the percentage of morphology of the water maze, respectively. (D) Correlation analysis between morphology and rotarod. (E–J) Correlation analysis between morphology and the number of food arm entries, duration, distance, number of wrong arm entries, duration, and distance in the Y‐maze. HI, hypoxia–ischemic. [Color figure can be viewed at wileyonlinelibrary.com]

To investigate whether there was a correlation between the impairment of morphology and behavioral performance, we used Pearson's correlation analysis to explore the correlation between behavior and morphology in HI neonatal rats. We used the percentage of dark neurons in total neurons in morphology and various parameters of MWM, Y‐maze, and rotarod for analysis. The results showed that there was a significant negative correlation between morphology and various indicators of water maze, rotarod, and Y‐maze (number and duration of food arm entries), with correlation coefficients ranging from 0.8694 to 0.6145, and the difference was statistically significant (Figure 10A–F, *p* < 0.01). However, the correlation of food arm distance was poor, with a correlation coefficient of 0.2123 (Figure 10G, *p* < 0.01). There was a significant positive correlation with the number, duration, and distance of Y‐maze error arm entries, with correlation coefficients ranging from 0.8264 to 0.7512, and the difference was statistically significant (Figure 10H–J, *p* < 0.01).

## 4 DISCUSSION

In this study, we found that inhalation anesthesia of 2% ISO for 1 h before the HI injury in neonatal rats significantly promoted neuronal regeneration, inhibited neuronal apoptosis, and improved motor and cognitive function, and ISO pretreatment inhibited neuronal cell death in the cerebral cortex, CA1, CA2, CA3, and DG of neonatal rats; that is, it improved acute and long‐term neurological deficits induced by HI brain damage in neonatal rats.

Previous studies have successfully established neonatal HI models using the Rice–Vannucci method^19^ and confirmed the degree of neurological and motor impairment of HI rats. In this study, we conducted a lot of behavioral experiments to determine the acute as well as long‐term neurological defects after HI injury. Among them, WMW and the Y‐maze tests were generally carried out to assess the learning and memory abilities,^20^, ^21^, ^22^ and the Rotarod test was used to observe the exercising ability and coordination function of rats.^23^, ^24^ Using these tests, we have found that the Morris‐water maze and Y‐maze tests can reflect a strong correspondence in terms of morphological damage and neurological changes, as demonstrated by significantly different behavioral performances in the tests. Behavioral evaluation can be used to assess long‐term behavioral changes in HIE rats. Behavioral assessment of rats 1 month after HI injury further determined the efficacy of ISO for long‐term neuroprotection. The experimental results showed that ISO reduced the severity of long‐term neurological injury, improved long‐term spatial learning and memory abilities, and increased motor coordination in HI rats. The HI group showed severe learning and memory impairment, and the littermates in the pretreatment group were not significantly different from those in the sham group, showing that ISO pretreatment can protect neurocognitive function. Our results are consistent with those of the study of Shao et al.^25^ Animals in the HI group showed more pronounced pathological damage to brain morphology, and these findings confirm previous studies showing a correlation between learning impairment and ipsilateral hemispheric and hippocampal tissue loss.^26^, ^27^

An increasing number of studies have shown that HI causes irreversible brain damage to the developing brain of newborns, resulting in long‐term neurological deficits.^28^, ^29^, ^30^ The hippocampus and the cortex are very sensitive to HI injury,^31^ so in this study, the above regions were selected to observe the morphological changes of neurons. At 24 h after HI, the neurons of the hippocampal CA1, CA3, and cortex in the HI group showed significant morphological damage, with loss of neurons and Nissl bodies, pyknotic nuclei, and vacuolization. The results of Nissl staining clearly showed that HIE induced extensive apoptosis in the ipsilateral hemisphere, which was effectively prevented by conditioning after ISO pretreatment through conditioned reflexes, consistent with the results of Galle et al.^32^ Our study further confirmed the neuroprotective effect of ISO pretreatment, which is also confirmed in the published literature.^33^ Pathologically, ISO pretreatment alleviated neuronal edema and necrosis, irregularly arranged Nissl bodies, incomplete cellularity, and inflammatory cell infiltration occurring in the cerebral cortex. The number of Nissl‐positive cells in the cortex and hippocampus increased after ISO pretreatment, indicating that ISO inhibits neuronal cell death in HI rats. In the study of Bauer et al.,^32^ it was confirmed that ISO postconditioning had a protective effect on HI brain damage in neonatal rats, and this protective effect may be related to the inhibition of the opening of the mitochondrial permeability transition pore (mPTP). Opening of mPTP can lead to impaired energy synthesis and cellular oxidative response, triggering a cascade, resulting in decreased adenosine triphosphate (ATP) levels, while the increase in the intracellular Ca^2+^ concentration is strongly linked to ATP levels.^32^, ^34^ It is generally accepted that the elimination ability is reduced by the massive release of glutamate during ischemia and hypoxia, the extracellular glutamate concentration is markedly increased to toxic levels, glutamate receptors are hyperactivated, and massive influx of Ca^2+^ leads to cell swelling and apoptosis, and this process is mainly mediated by AMPA receptors.^35^ It has been documented that administration of AMPA receptor antagonists to neonatal rats with HI brain damage alone can reduce the inflammatory response and peroxide levels in brain cells and reduce the degree of brain injury.^34^ It has also been suggested that the neuroprotective mechanism of hypoxic preconditioning is related to the downregulation of DNMT3A and DNMT3B levels in DNA methyltransferase lines (DNMTs), maintenance of hippocampal neuronal cell viability, and inhibition of apoptosis.^36^ The excitatory neurotransmitter AMPA, on the other hand, activates AMPA receptors and causes changes in their configuration, resulting in damaging effects, and it has also been shown to cause brain tissue damage.

Through correlation analysis between behavior and morphology, we found that there was a strong correlation between behavioral performance and dark neuron, with the less dark neuron the batter the behavioral performance of the animal. Thus, the behavioral difference between WMW and Y‐maze can be used as a behavioral assessment reference for brain injury.^22^, ^37^ All these results reveal that ISO preconditioning can improve spatial learning and memory abilities, as well as long‐term motor function with positive effects.

ISO is an inhalation anesthetic with certain analgesic and muscle relaxant effects. There is no contraindication for its use. It is applicable to various types of intraoperative anesthesia. It has a close relationship with the biological behavior of malignant tumor histiocytes such as colorectal cancer, prostate cancer, and glioma.^38^, ^39^ A large number of studies have reported that low‐dose ISO has antioxidant, antiapoptotic, and immunomodulatory effects, and has a good protective effect on various acute central nervous system injuries such as cerebral ischemia–hypoxia injury and reperfusion injury.^40^ In addition, some studies have confirmed that ISO can regulate the activation of the NF‐κB signaling pathway,^41^, ^42^ and ISO or multiple treatments during development can affect spatial cognitive behavior in rats as adults.^43^ Previous studies^44^, ^45^ have shown that ISO may contribute to postoperative cognitive dysfunction in elderly patients by eliciting neuroinflammation, disrupting choline function, and synaptic plasticity. It can induce postoperative cognitive dysfunction and promote neuronal apoptosis. Recent studies^46^ have shown that ISO can alleviate the inflammatory factor analysis and the degree of lipid peroxidation in rats with cerebral ischemia/reperfusion injury through the TGF‐β/Smad2/3 signaling pathway, reduce the hydrolytic activity of matrix metalloproteinases in brain tissue, reduce tight junction protein loss, and improve cerebral ischemia–reperfusion injury in rats. The in vitro studies^47^ demonstrated that ISO preconditioning reduced the release of oxygen‐glucose deprivation (OGD)‐induced lactate dehydrogenase (LDH) and increased OGD‐inhibited cell viability. It has also been observed that the hypoxia‐inducible factor‐1α (HIF‐1α) was increased under ISO preconditioning. In fact, these results thus suggest that ISO preconditioning may provide potential neuroprotection against ischemic/reperfusion injury by upregulating HIF‐1α expression through Akt/mTOR/s6K activation. McAuliffe et al.^48^ studied the long‐term effects of delayed preconditioning with ISO, hypoxia, or room air on motor and cognitive functions in mice subjected to HI for 65 min at postnatal Day 10. Delayed preconditioning of ISO and hypoxia in neonatal mice improved learning and memory with functional neuroprotection. The study of Xiong et al.^49^ demonstrated that repeated ISO anesthesia induces ischemic tolerance in rats in a dose–response manner. Globulin (GLB), an adenosine triphosphate‐regulated potassium channel blocker, abolished the tolerance induced by ISO. Brief ISO anesthesia induces ischemic tolerance in the brain. The effect was found to be dose‐dependent in a rat focal cerebral ischemia model. Ischemic tolerance induced by ISO preconditioning is dependent on the activation of adenosine triphosphate‐regulated potassium channels.

HIE is a common neonatal disease in clinical practice at present, and the pathogenesis includes mitochondrial damage, oxidative stress, neurotoxicity of excitatory amino acids, and inflammatory immune response due to a combination of cellular and molecular mechanisms. Its occurrence is mainly related to perinatal asphyxia and plays an indispensable role in perinatal neurological diseases, which can cause brain tissue hypoxia, interruption or reduction of cerebral blood flow, brain injury in newborns, neurological injury, and even death in children.^50^, ^51^ At present, mild hypothermia therapy is often used to treat moderate and severe full‐term HIE children in clinical practice, which has a regulatory effect on the body's cerebral blood flow and can reduce neuronal apoptosis and relieve neurological symptoms such as disturbance of consciousness and floppy limbs in children.^52^ It is undeniable that hypothermia treatment is recognized as an effective treatment for HIE.^53^, ^54^ However, its clinical application is limited due to the short treatment window and other reasons. In addition, the pathogenesis and clinical characteristics of HIE are complex. Current studies have shown that drugs combined with hypothermia are more effective in the treatment of HIE.^55^, ^56^, ^57^ To make the neuroprotective effect of HIE more clinically applicable, combined hypothermia treatment needs to be further considered and explored to achieve more targets and enhance neuroprotection. There is a lack of drugs for the treatment of HIE in clinical practice, and there is still an urgent need to find safe and effective drugs from natural products.

## 5 CONCLUSION

Taken together, we found that the ISO pretreatment not only protected HI neonatal rats from brain injury but also promoted their neurological recovery and improved learning and memory, possibly by inhibiting cell death in the cortex and hippocampus after HI. After ISO preconditioning, reduce brain injury and improve neurological function. Notably, the specific mechanism of the protective effect of ISO pretreatment has not been fully clarified. We speculate that this is achieved by inhibiting cell death in the cerebral cortex and the hippocampus after HI, and we will further study this in depth.

## AUTHOR CONTRIBUTIONS

Yi‐Bo Wang participated in the design and review of this study. Yi Fei‐Sun carried out the experiment. Hao‐Yue Qin, Senio Campos de SouzaHan, and Han Xue interpreted the data and arranged figures. Miao Huang, Yu‐Ying Wang, and Yi‐Bo Wang wrote and revised the manuscript. All authors have read and approved the final version of the manuscript.

## CONFLICT OF INTEREST

The authors declare no conflict of interest.

## ETHICS STATEMENT

This study was approved by the Ethics Committee of Kunming Medical University (reference number: kmmu 20220748). The experimental protocol was established according to the ethical guidelines of the Declaration of Helsinki, in accordance with the ARRIVE guidelines in terms of ethical approval and consent to participate, and all methods were performed in accordance with relevant guidelines and regulations.

## TRANSPARENCY STATEMENT

All the authors affirm that this manuscript is an honest, accurate, and transparent account of the study being reported; that no important aspects of the study have been omitted; and that any discrepancies from the study as planned (and, if relevant, registered) have been explained.

## Data Availability

The data presented in this study are available on request from the corresponding author.
