# Correction to “Protective effect of isoflurane preconditioning on neurological function in rats with HIE”

**DOI:** 10.1002/ibra.12142

**Published:** 2023-12-04

Sun Y‐F, Huang M, Qin H‐Y, et al. Protective effect of isoflurane preconditioning on neurological function in rats with HIE. *ibrain*. 2022;8:500‐515. doi:10.1002/ibra.12081

In ETHICS STATEMENT section, “This study was approved by the Ethics Committee of Kunming Medical University (reference number: kmmu 20220748). The experimental protocol was established according to the ethical guidelines of the Declaration of Helsinki, in accordance with the ARRIVE guidelines in terms of ethical approval and consent to participate, and all methods were performed in accordance with relevant guidelines and regulations.” was inaccurate.

This statement should be corrected as “This study was approved by the Ethics Committee of Kunming Medical University (reference number: kmmu 20220748).”

We apologize for this error.

